# Regional beat-to-beat variability of repolarization increases during ischemia and predicts imminent arrhythmias in a pig model of myocardial infarction

**DOI:** 10.1152/ajpheart.00732.2022

**Published:** 2023-05-05

**Authors:** Matthew Amoni, Sebastian Ingelaere, Jonathan Moeyersons, Dries Wets, Aldo Tanushi, Sabine Van Huffel, Carolina Varon, Karin Sipido, Piet Claus, Rik Willems

**Affiliations:** ^1^Experimental Cardiology, Department of Cardiovascular Sciences, KU Leuven, Leuven, Belgium; ^2^Department of Cardiology, University Hospitals, Leuven, Belgium; ^3^Department of Electrical Engineering, STADIUS Center for Dynamical Systems, Signal Processing and Data Analytics, KU Leuven, Leuven, Belgium; ^4^Microgravity Research Center, Université Libre de Bruxelles, Brussels, Belgium; ^5^Cardiovascular Imaging and Dynamics, Department of Cardiovascular Sciences, KU Leuven, Leuven, Belgium

**Keywords:** acute myocardial ischemia, beat-to-beat variability of repolarization, myocardial infarction, sudden cardiac death, ventricular fibrillation

## Abstract

Ventricular arrhythmia (VT/VF) can complicate acute myocardial ischemia (AMI). Regional instability of repolarization during AMI contributes to the substrate for VT/VF. Beat-to-beat variability of repolarization (BVR), a measure of repolarization lability increases during AMI. We hypothesized that its surge precedes VT/VF. We studied the spatial and temporal changes in BVR in relation to VT/VF during AMI. In 24 pigs, BVR was quantified on 12-lead electrocardiogram recorded at a sampling rate of 1 kHz. AMI was induced in 16 pigs by percutaneous coronary artery occlusion (MI), whereas 8 underwent sham operation (sham). Changes in BVR were assessed at 5 min after occlusion, 5 and 1 min pre-VF in animals that developed VF, and matched time points in pigs without VF. Serum troponin and ST deviation were measured. After 1 mo, magnetic resonance imaging and VT induction by programmed electrical stimulation were performed. During AMI, BVR increased significantly in inferior-lateral leads correlating with ST deviation and troponin increase. BVR was maximal 1 min pre-VF (3.78 ± 1.36 vs. 5 min pre-VF, 1.67 ± 1.56, *P* < 0.0001). After 1 mo, BVR was higher in MI than in sham and correlated with the infarct size (1.43 ± 0.50 vs. 0.57 ± 0.30, *P* = 0.009). VT was inducible in all MI animals and the ease of induction correlated with BVR. BVR increased during AMI and temporal BVR changes predicted imminent VT/VF, supporting a possible role in monitoring and early warning systems. BVR correlated to arrhythmia vulnerability suggesting utility in risk stratification post-AMI.

**NEW & NOTEWORTHY** The key finding of this study is that BVR increases during AMI and surges before ventricular arrhythmia onset. This suggests that monitoring BVR may be useful for monitoring the risk of VF during and after AMI in the coronary care unit settings. Beyond this, monitoring BVR may have value in cardiac implantable devices or wearables.

## INTRODUCTION

Sudden cardiac death (SCD) is a leading cause of death and occurs predominantly because of unexpected ventricular tachycardia (VT) and ventricular fibrillation (VF) with subsequent sudden cardiac arrest (1). The majority of SCDs occur in patients with ischemic heart disease (IHD) and are often the first presentation of acute myocardial ischemia (AMI) (2). Several factors contribute to the occurrence of an AMI including the characteristics of the coronary lesion, particularly thin-walled atherosclerotic plaques, and hypercoagulability (3). However, the vulnerability to SCD during AMI and factors predicting SCD as a manifestation of AMI remain under investigation (4). Structural elements such as the severity and extent of the infarcted area, cellular electrophysiological changes during AMI, and sympathetic hyperresponsiveness promote SCD (5). Identifying and monitoring electrophysiological factors that predict imminent arrhythmia can provide a better identification of patients at risk and an opportunity to prevent SCD via monitoring wearables and early warning systems (6).

The timing and prediction of SCD in patients with AMI are poorly defined because it is difficult to capture extensive data of these unexpected events. Insights are gained from preclinical animal models, where lethal arrhythmias occur early in AMI during *phase I* (<30 min) and fewer occur in *phase II* (1–24 h) (7, 8). Early arrhythmias can be “immediate” (*phase 1a*), within 10 min, or “delayed” (*phase 1b*), within 12–30 min of AMI, when most arrhythmias occur (8). The principal mechanism is functional reentry and involves progressive cell uncoupling and reduced membrane potential with electrical propagation and repolarization abnormalities in the infarct and border zone leading to unidirectional block and rotors (9). It would be of interest to understand why some individuals are vulnerable to SCD during this time window and to predict the occurrence of these arrhythmias preferably by noninvasive means, to perform novel or more strict primary prevention of sudden death in atherosclerotic heart disease, and possibly to offer monitoring with future wearable technology in these individuals.

Short-term QT interval variability (STVQT), here referred to as beat-to-beat variability of repolarization (BVR), is one measure of QT interval variability (10). BVR has been shown to be an electrocardiographic predictor of imminent arrhythmia in the dog model of Torsades de Pointes during proarrhythmic drug infusion (11, 12). Temporal changes in BVR have been recently proposed for the prediction of nonsustained ventricular tachycardia (nsVT) in animal models of and patients with ischemic heart disease at risk of SCD (13–15). This short-term measure of QT interval variability moreover has the potential to be built into implantable defibrillators to automatically detect changes in BVR and activate preventive pacing to prevent ventricular arrhythmias (13). During AMI, BVR and its predictive value for SCD have not been evaluated. However, repolarization abnormalities including increased BVR (16), QT interval variability indices in 5-min epochs or 1-h epochs (17, 18), T-wave alternans (TWA) (19), QT dispersion (20), and non-TWA repolarization variability (21) have been previously shown to occur during and after myocardial ischemia. Data from intracardiac monitoring of monophasic action potential (MAP) during AMI demonstrate increased BVR (22) during AMI, although its relationship to SCD was not evaluated.

Therefore, we hypothesized that BVR increases during AMI and is detectable on the surface electrocardiogram (ECG). Furthermore, we posit BVR exhibits a spatial pattern with higher indices in leads directed to the vulnerable territory of regional ischemia. In this study, we used a pig model of AMI to study ECG-BVR. We studied the evolution of BVR during AMI and its relation to imminent SCD events, and we evaluated BVR in the chronic phase after AMI and assessed its correlation to the inducibility of ventricular arrhythmia.

## MATERIALS AND METHODS

Domestic pigs (*n* = 24; Strain TN70, Topigs, Norsvin) of both sexes were used in this study. Animal husbandry was in accordance with international guidelines (European Directive 2010/63/EU), and protocols were approved by the KU Leuven institutional ethical committee (ECD137/2018).

All experiments were performed under general anesthesia with mechanical ventilation. Animals were sedated with tiletamine-zolazepam (8 mg/kg im) and xylazine (2.5 mg/kg im). Anesthesia was induced with propofol (3 mg/kg iv) and maintained (10 mg/kg/h iv), combined with remifentanil (0.2–0.4 µg/kg/h iv). Endotracheal ventilation was performed at 6–10 mL/kg with 50% oxygen-air mixture. Antibiotics [preoperative cefazolin (25–50 mg/kg iv) and postoperative amoxicillin (15 mg/kg or enrofloxacilin 4 mg/kg im) and anticoagulation (acetylsalicylic acid 500 mg iv) and periodic heparin (5,000–10,000 IU iv)] were routinely administered during procedures.

### Pig Model of Acute Myocardial Ischemia

**Figure 1.**
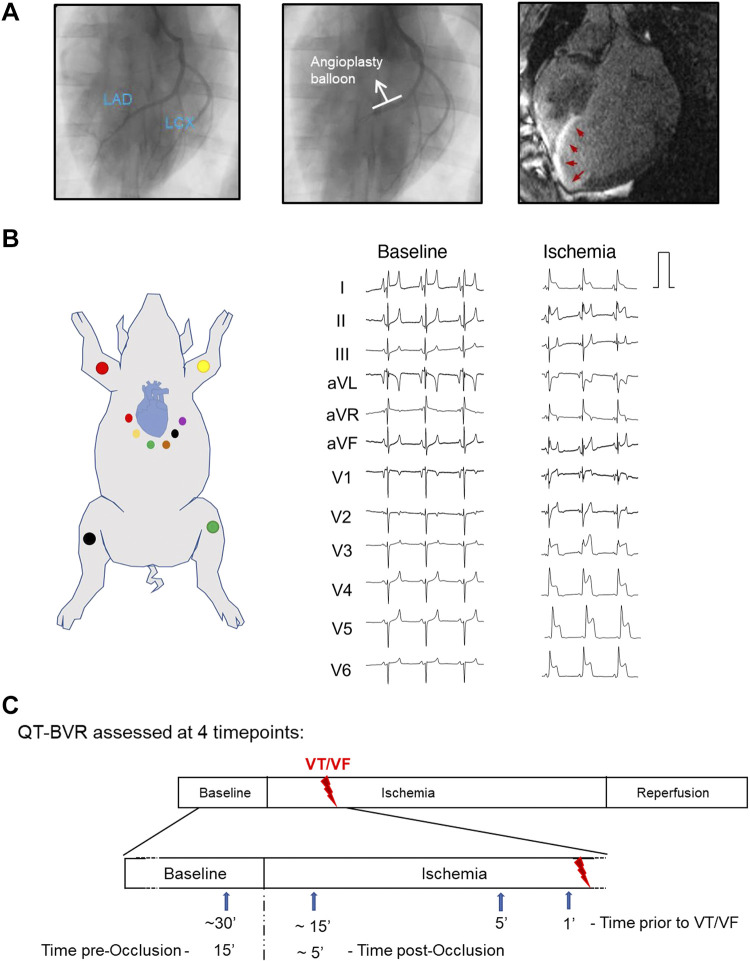
Assessment of beat-to-beat variation of repolarization (BVR) in a pig model of myocardial infarction and sudden cardiac death. *A*: anterior-posterior angiographic views of the left coronary arteries (*left*) illustrating occlusion of the mid left anterior descending artery (LAD; *middle*). *A*, *right*: cardiac magnetic resonance imaging of the infarcted heart 1 mo after ischemia-reperfusion (I/R) illustrating 21% infarction of left ventricular mass. Red arrows indicate infarct scar. *B*: schematic illustrating the ECG electrode placement in the pig (*left*) and examples of the 12-lead ECG at baseline and during ischemia (*right*); note the dramatic ST changes in V1–V6. *C*: illustration of the experimental design and selected taken to analyze BVR. aVF, augmented vector foot; aVL, augmented vector left; aVR, augmented vector right; ECG, electrocardiogram; LCX, left circumflex coronary artery; VT/VF, ventricular arrhythmia; *n*, number of animals.

All animals were premedicated with 300-mg oral amiodarone for 3 days, as well as 300-mg aspirin and clopidogrel 300 mg for 1 day. AMI was induced by balloon occlusion of the left anterior descending (LAD) coronary artery in 16 animals as previously described (MI) (23). Briefly, the right carotid artery was dissected and cannulated with a 6/7-Fr vascular sheath. A 6/7-Fr Judkins left coronary guiding catheter was positioned in the left main coronary ostium under fluoroscopic guidance. A 2.5–3.0-mm angioplasty balloon was completely inflated in the left anterior descending (LAD) immediately after the first diagonal branch, resulting in anterior-septal ischemia (Fig. 1*A*). Occlusion was maintained for 120 min, followed by release and reperfusion with 60 min monitoring under anesthesia. In the event of sustained VT/VF, external defibrillation with a shock of 150–200 J was applied to restore sinus rhythm. Eight sham-operated animals were included as controls (sham). Animals were randomly assigned to either MI or sham. No animals died during the procedure. Postoperatively, animals received broad-spectrum antibiotics (15 mg/kg amoxicillin or 4 mg/kg enrofloxacilin) with analgesia, Buprenorphine-HCl (0.024 mg/kg–0.03 mg/kg im). All animals received amiodarone for 3 days. Aspirin (100 mg) and clopidogrel (75 mg) were given daily for 1 mo.

### Electrocardiogram Recording and Analysis

Baseline was defined as a 1-min segment, 15 min before coronary occlusion. For assessment of the spatial changes in BVR during AMI, a 1-min segment during early ischemia (5 min postocclusion) from all leads of the 1 kHz, 12-lead ECG was compared with baseline. To assess the BVR changes preceding VT/VF, animals subjected to AMI were subdivided into two groups: those that developed VF (VF +ve), *n* = 10, and those without VF (VF −ve), *n* = 6. The first episode of VF was annotated and two time points: 5 and 1 min before the spontaneous VF were defined (Fig. 1*C*). VF −ve animals were matched by weight and sex to 6 VF +ve animals, and individually matched time points from the start of coronary occlusion were used to define the 5- and 1-min pre-VF time points. A 1-min ECG segment at these time points was used for analysis. ECG segments were coded to blind the investigator and analyzed using R-DECO: a modified fiducial point detection custom-made MATLAB software (24). We employed a semiautomated fiducial segment averaging template-based method (25). Our prior assessment showed that this was the most consistently accurate method for QT detection (26). Correct annotation of fiducial points was checked and manually adjusted where necessary using a tangent method. Repolarization lability in the ECG QT-BVR was then calculated using: $\text{Σ}(|{\text{QT}}_{n+1}-{\text{ QT}}_{n}|)/[n\text{ beats }\times \;\surd 2]$ (11).

Premature ventricular complexes (PVCs) were counted in 5-min intervals within the 30 min after occlusion and documented. As demanded and previously described, all ventricular premature complexes as well as the preceding (if the premature complex occurred before the end of the T wave) and the succeeding sinus beats were excluded from the analysis (10, 14). The underlying rhythm and the presence of premature complexes were ascertained by several coauthors with medical background (MA, DW, and AT).

### Sampling Rate

The impact of sampling frequency on other measurements of QT variability in a set of metrics in the time and frequency domain has already been studied and it was shown that a sampling rate of >500 Hz is necessary for reliable measurements (27). To assess the impact of sampling frequency and recording systems specifically on BVR, three sets of ECGs were simultaneously recorded at 200 Hz (Holter device; Microport, Belgium), 1 kHz (Powerlab system; ADInstruments, UK), and 4 kHz (Labsystem Pro electrophysiology system; BARD, Boston Scientific). All systems employed a 50-Hz lowpass filter. The ECG electrodes were positioned to mimic the clinical ECG, and precordial placement was modified to adapt to the orientation of the heart in the pig (Fig. 1*B*). All recordings were exported and analyzed on an offline workstation. To assess the impact of sampling frequency on BVR assessment, lead A of Holter recordings and equivalent lead II of 12-lead ECG were used at baseline.

Supplemental Figure S1*A* illustrates the ECG analysis and QT-interval detection methods for BVR calculation and visualization (all Supplemental material is available at https://doi.org/10.48804/QT0K2J). At baseline, BVR of ECGs recorded at 200 Hz was significantly higher than the BVR simultaneously recorded at 1 kHz (200 Hz, 2.02 ± 0.72 ms vs. 1 kHz, 0.31 ± 0.18 ms, *P* < 0.0001). The magnitude of BVR recorded at 1 kHz was comparable to the 4 kHz ECG (1 kHz, 0.31 ± 0.18 ms vs. 4 kHz, 0.36 ± 0.21 ms, *P* = 0.9800; Supplemental Fig. S1, *B* and *C*). To investigate whether the lower sampling frequency indeed produced higher BVR, we downsampled the 1 kHz baseline signal to 200 Hz (Supplemental Fig. S2). This reproduced the increased BVR magnitude observed in the 200-Hz signal recorded by the Holter.

There was no difference between MI and sham BVR at baseline in both the low-sampling 200 Hz and high-sampling 1 and 4 kHz signals. These findings demonstrate that also BVR magnitude is influenced by the sampling frequency and reinforces the requirement of uniform sampling frequency for comparison. Therefore, a sampling frequency of 1 kHz was used for analysis of the data.

### Serum Biomarker Assessment

Serum troponin and creatinine kinase were determined before and during AMI, as well as 1-mo postprocedure, as a surrogate of severity of myocardial injury. Blood was collected at baseline, defined as a time at least 15 min before occlusion, as well as at the end of 120 min of ischemia, using EDTA and Li-heparin tubes. Blood samples were analyzed by the routine clinical assays of the clinical laboratory of the university hospital Leuven.

### Cardiac Magnetic Resonance Imaging

After 1 mo, cardiac dimensions and function as well as left ventricle (LV) infarct size were assessed by magnetic resonance imaging (MRI) under general anesthesia with artificial ventilation in 19 of the 24 pigs, 12 MI and 7 sham [2 MI pigs succumbed to SCD in the 1st wk after MI induction and 3 animals, 1 sham and 2 MI, had high heart rates (>150 beats/min) not suitable for imaging]. The protocol of cMRI performed with a 3 T, PRISMA-tim whole body scanner (Siemens, Erlangen, Germany) has been previously described (28). Infarct size was determined by late gadolinium enhancement (LGE), 12 min following a double dose bolus of Gd (gadoterate meglumine, Dotarem, 0.2 mmol/kg). Structural and functional assessment parameters are summarized in Table 1.

**Table 1.** Cardiac magnetic resonance imaging data of structural and functional remodeling 1 mo after MI

	MI	Sham	*P* Value
Pigs, *n*	12	7	
Infarct size, %	19.3 ± 4.2	0 ± 0	<0.0001
Ejection fraction, %	38.5 ± 10.3	62.3 ± 3.2	<0.0001
End-diastolic volume, mL	170.7 ± 22.6	142 ± 24.7	0.0014
End-systolic volume, mL	104.9 ± 25.9	51.0 ± 10.1	<0.0001
LV mass, g	132.1 ± 17.7	133.9 ± 24.5	0.1298
Heart rate, beats/min	81.6 ± 10.8	74 ± 16.9	0.0876
LV peak systolic pressure, mmHg	94.3 ± 12.2	103.0 ± 18.2	0.1949
LV mean diastolic pressure, mmHg	5.7 ± 4.7	1.5 ± 1.4	0.0184

Values are means ± SD. LV, left ventricular; MI, myocardial infarction.

### Cardiac Electrophysiology Study

Two to four days after the cardiac MRI, a follow-up in vivo electrophysiology (EP) study was performed in a subset of 15 pigs (9 MI and 6 sham). Under general anesthesia, a 12-lead ECG was recorded at 1 kHz as described previously for at least 5 min. Then, the femoral vein, jugular vein, and carotid artery were cannulated with 7–8-Fr vascular sheaths. Standard EP catheters (Decanav Biosense Webster, Belgium) were positioned in the LV, right ventricle (RV), and coronary sinus as previously described (28). One MI pig was excluded because of a vascular access complication. A ventricular arrhythmia induction protocol was performed by programmed electrical stimulation (PES) from the RV apex (adapted from Refs. 16 and 29) in eight MI and six sham. To avoid induction of a nonspecific VF, stimulation in the LV and burst pacing were not done. Animals without arrhythmias in this single-site protocol were considered noninducible.

The inducibility of VT/VF as a surrogate of arrhythmia vulnerability was assessed using a scale constructed from the stepwise progression of the PES protocol where the first step (S1 = 600 ms and 1 extrasystole, S2 = 400 ms) was 100% and the last most aggressive step (S1 = 350 ms and 3 extrasystoles, S2 = 200 ms, S3 = 180 ms, S4 = 150 ms) marked noninducibility as 0%, adapted from León et al. (29).

### Statistical Analysis

All data are presented as means ± SD or number (percentage). Distribution of data was tested by Shapiro–Wilk. Data were analyzed with Student’s *t* test or Mann–Whitney test, one- or two-way mixed-effects model ANOVA with Bonferroni post hoc testing or Kruskal–Wallis test with Dunn’s post hoc testing, Fisher’s exact test, and linear regression analysis as applicable. *P* < 0.05 was considered statistically significant.

## RESULTS

### BVR Increases During Regional Ischemia Proportional to the Extent of Injury

**Figure 2.**
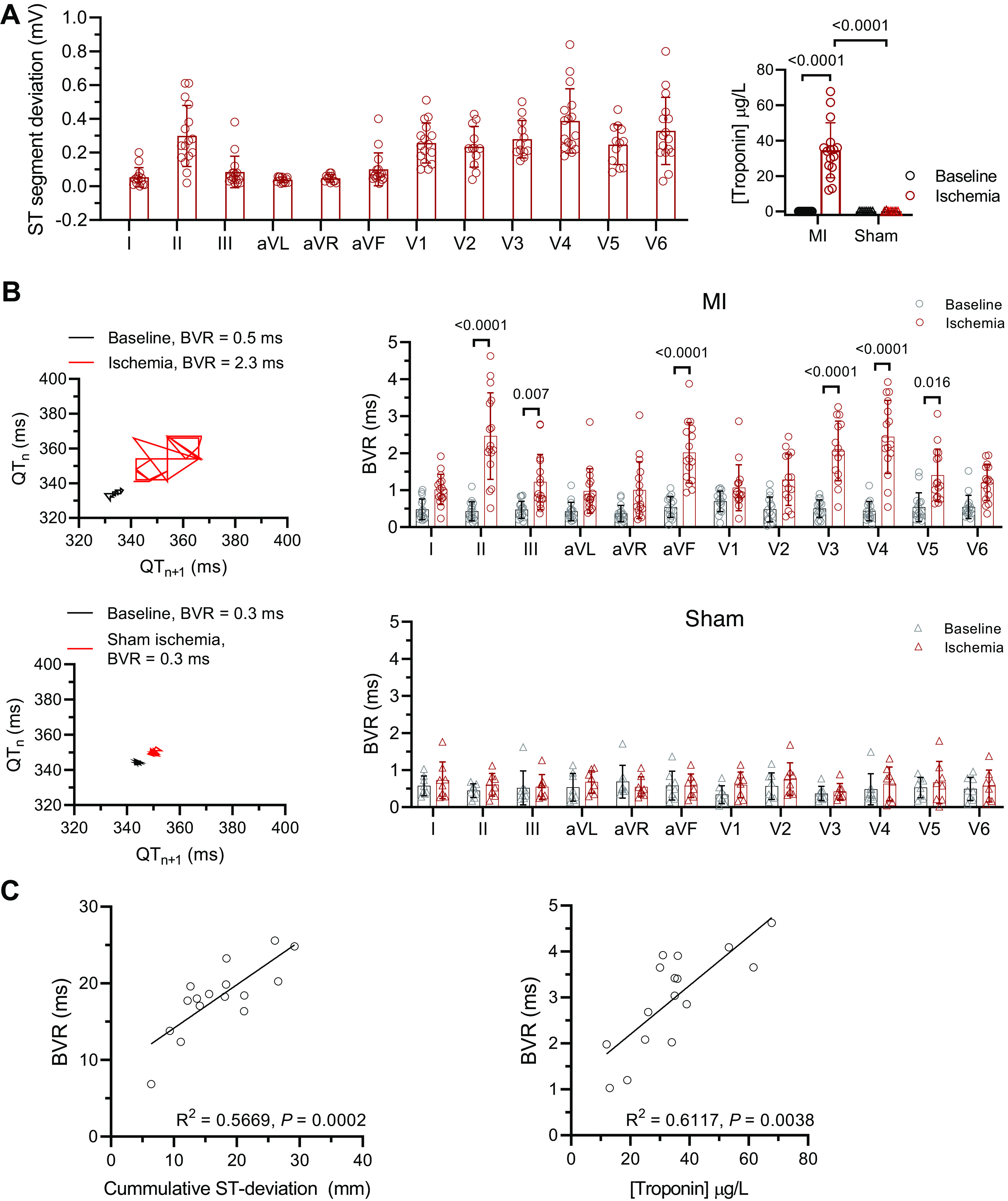
Beat-to-beat variation of repolarization (BVR) increases during regional ischemia and is related to the extent of injury. *A*: summary data of 12-lead ECG ST-segment deviation (*left*) and serum troponin after 120 min acute myocardial ischemia (*right*); *n* = 16 myocardial infarction (MI) and 8 sham. One-way and mixed-effects model ANOVA with Bonferroni posttest, respectively. *B*, *left*: example of Poincaré plots of the QT interval at baseline and during ischemia in MI (*top*) and sham (*bottom*) animals. *B*, *right*: summary data of 12-lead ECG BVR; *n* = 16 MI and 8 sham. Mixed-effects model ANOVA with Bonferroni posttest. *C*, *left*: correlation between cumulative BVR and ST deviation in all 12 ECG leads. *C*, *right*: correlation between cumulative BVR and serum troponin; *n* = 16 MI. Linear regression analysis. ANOVA, analysis of variance; aVF, augmented vector foot; aVL, augmented vector left; aVR, augmented vector right; ECG, electrocardiogram; *n*, number of animals.

We investigated the impact of AMI on BVR. During AMI, the ST segment deviated from baseline in all leads, mean absolute deviation of 0.23 ± 0.15 mV and was largest in *leads II* and *V4*, consistent with the anterior-septal AMI (Fig. 2*A*). The serum troponin rose significantly (AMI, 34.60 ± 15.54 μg/L vs. baseline, 0.01 ± 0.01 μg/L, *P* < 0.0001), but only correlated weakly with infarct size (*R*^2^ = 0.56, *P* = 0.003), possibly reflecting the discrepancy between the sampling time, i.e., serum sampling immediately after MI compared with the imaging after 1 mo with additional reperfusion injury and remodeling in between the time points. Figure 2*B* presents BVR changes on the 12-lead ECG during AMI. BVR increased significantly in leads covering the territory of injury including II, III, aVF, V3, V4, and V5, whereas sham animals showed no significant changes at matched time points.

**Figure 3.**
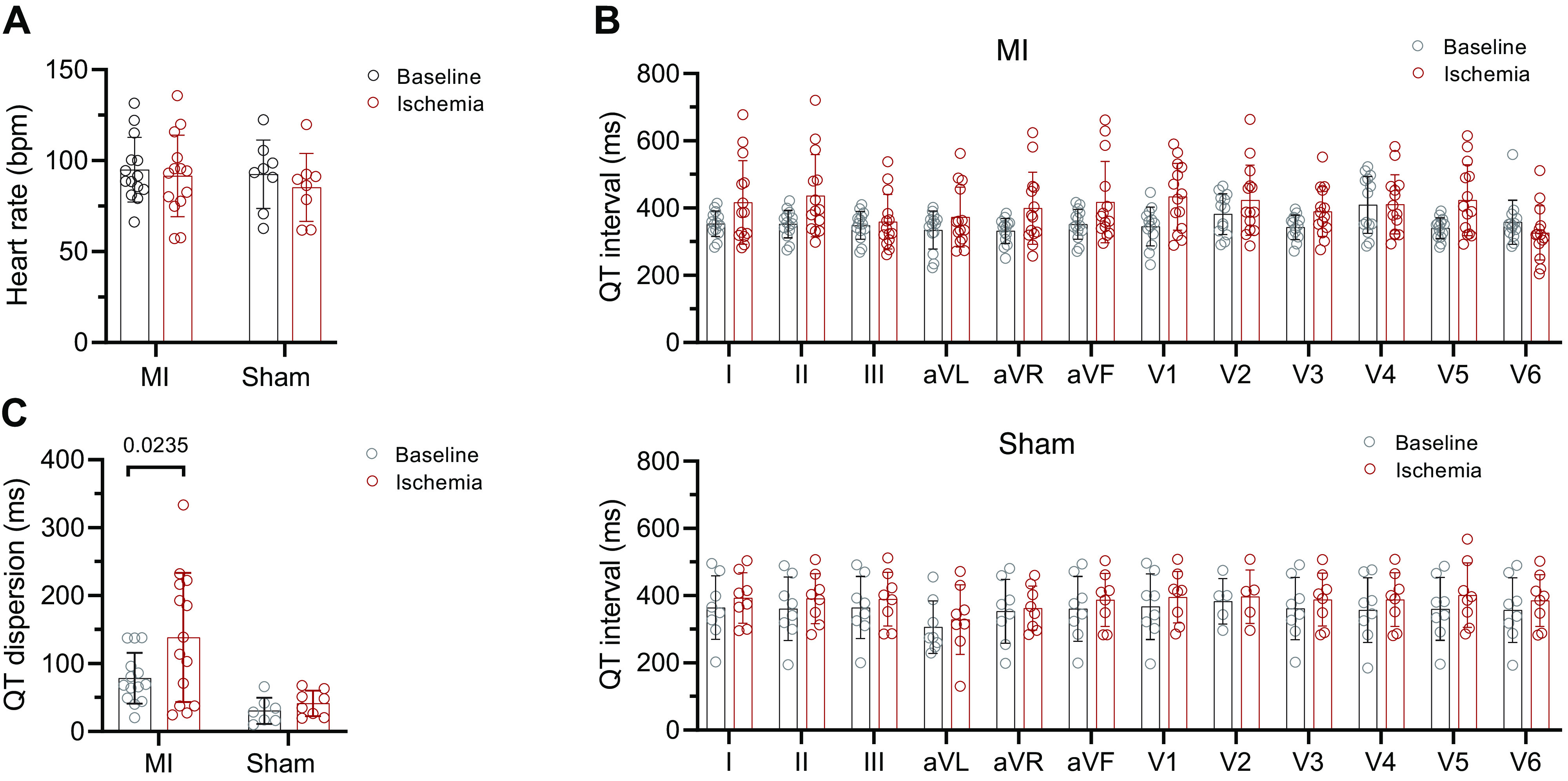
Heart rate and QT interval during ischemia. *A*: summary data of heart rate [in beats/min, (bpm)] at baseline and during ischemia. *B*: summary data of QT interval duration in the 12 ECG leads at baseline and during ischemia in myocardial infarction (MI; *top*) and sham (*bottom*). *C*: summary data of QT dispersion at baseline and during ischemia; *n* = 16 MI and 8 sham. Mixed-effects model ANOVA with Bonferroni posttest. ANOVA, analysis of variance; aVF, augmented vector foot; aVL, augmented vector left; aVR, augmented vector right; ECG, electrocardiogram; *n*, number of animals.

The cumulative BVR correlated with the cumulative ST-segment deviation of all leads (*R*^2^ = 0.567, *P* = 0.0002) (Fig. 2*C*), and *leads II* and *V4* had the highest correlations (Supplemental Fig. S3). The maximal BVR in each pig also correlated with the serum troponin levels (*R*^2^ = 0.612, *P* = 0.0038) (Fig. 2*C*). There was no significant change in heart rate between baseline and AMI, or between MI and sham (Fig. 2*A*). Although the QT duration was not different between baseline and AMI, the QT dispersion increased during ischemia (Fig. 3). Together, these data suggest that BVR increases during acute ischemia in keeping with the region and extent of injury.

### BVR Increases Before Spontaneous VT/VF During Ischemia

**Figure 4.**
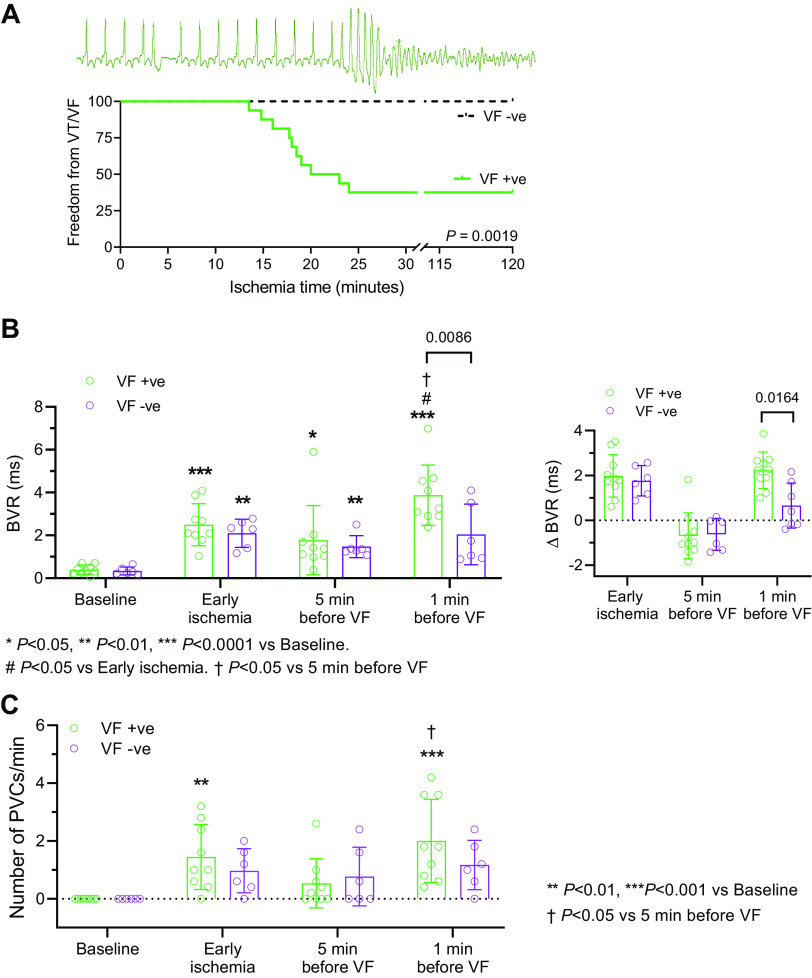
Beat-to-beat variation of repolarization (BVR) increases further before spontaneous ventricular fibrillation (VF) during ischemia. *A*: typical example of spontaneous onset VF during acute myocardial ischemia (AMI; *top*), and Kaplan–Meier plots of freedom from ventricular arrhythmia (VT/VF) during AMI. Log-rank test. *B*, *left*: summary data of temporal BVR changes during AMI in relation to the occurrence of VF and matched time points (by duration of ischemia) in six sex- and weight-matched animals without VF. *B*, *right*: summary data of the change in BVR from *B*, *left*; *n* = 10 animals with (VF +ve) and 6 animals without (VF −ve) VF. Multilevel mixed-effects linear model with Bonferroni correction. *C*: summary data of the incidence of premature ventricular complexes (PVCs) during AMI; *n* = 10 VF +ve and 6 VF −ve. Multilevel mixed-effects linear model with Bonferroni correction. VT, ventricular tachycardia; *n*, number of animals.

We assessed the behavior of BVR before spontaneous onset VF in our model of AMI in *lead II* or *V4* as appropriate. Figure 4*A* shows a typical example of a spontaneous onset VF. VF occurred in 72% of cases at 15–23 min after AMI. Figure 4*B* presents the changes in BVR in VF +ve and VF −ve animals during AMI. In VF +ve cases, BVR at 5 min before VF was comparable to BVR in early ischemia, 5 min AMI (5 min pre-VF, 1.67 ± 1.56 ms vs. 5 min AMI, 2.49 ± 0.98 ms, *P* = 0.3468). In the minute before VF, BVR increased significantly (3.78 ± 1.36 ms vs. 5 min pre-VF, 1.67 ± 1.56 ms, *P* < 0.0001). In animals that did not have VF (VF −ve), BVR changes were assessed using a matched time point during AMI, BVR did not increase significantly between the matched time points during AMI (2.04 ± 1.42 ms vs. 5 min prematched, 1.47 ± 0.51 ms, *P* = 0.5926) and compared with early ischemia (vs. 5 min AMI, 2.10 ± 0.66 ms, *P* = 0.7997). Thus, after a similar duration of ischemia (17.5 ± 5.4 min, corresponding to the 1 min before VF), a significant difference was observed in the change in BVR between the animals that developed VF (VF +ve) and the ones that did not (VF −ve) (2.23 ± 0.81 ms vs. VF −ve, 0.66 ± 1.00 ms, *P* = 0.0164; Fig. 4*B*). Furthermore, in sham animals, no temporal changes in BVR were observed at matched time points (Supplemental Fig. S4). These data suggest that BVR changes can be a predictor of imminent VF during AMI. There was no difference in the evolution of BVR during ischemia between male or female pigs.

We also examined the occurrence of premature ventricular complexes (PVCs) that could provide the triggers that initiate VF. Figure 4*C* shows the temporal incidence of PVCs, which exhibited a similar pattern of temporal behavior as BVR. The coupling interval of the PVC that initiated VF and PVCs in the 1 min before VF was similar and short (218 ± 28 ms vs. 1 min pre-VF, 225 ± 46 ms, *P* = 0.9122), but tended to be shorter than PVCs observed in the early ischemic period (5 min AMI, 302 ± 96 ms, *P* = 0.0865). These observations highlight the possible importance of PVC timing and an evolution during MI until the ideal timing and substrate coincide to initiate and sustain VF.

The electrocardiographic and clinical parameters of severity of injury during AMI comparing VF +ve and VF −ve animals are presented in Table 2. The maximum frequency of PVCs was higher in VF +ve animals (2.5 ± 1.0/min vs. 1.4 ± 0.6/min in VF– ve; *P* = 0.025), and serum troponin levels tended to be greater (35.8 ± 14.2 μg/L vs. VF – ve, 21.3 ± 9.9 μg/L, *P* = 0.0780). However, other parameters including heart rate, QT interval, ΔQT, and QT dispersion were not greater in VF +ve animals, suggesting the extent or severity of injury may contribute to arrhythmogenesis.

**Table 2.** Electrocardiographic and AMI injury characteristics of VF + ve and VF − ve animals

	VF +ve	VF −ve	*P* Value
Pigs, *n*	10	6	
ST-segment deviation, mm	5.2 ± 2.0	3.8 ± 1.2	0.1102
Heart rate, beats/min	89.2 ± 20.9	98.4 ± 24.5	0.4608
QT, ms	458.8 ± 137.9	457.5 ± 89.9	0.9845
QT dispersion, ms	161.4 ± 94.0	129.2 ± 88.7	0.5020
Maximal PVC frequency, PVCs/min	2.5 ± 1.0	1.4 ± 0.6	0.0250
Serum troponin, μg/L	35.9 ± 14.2	21.3 ± 9.9	0.0708
Infarct size, %LV mass	20.4 ± 5.0	17.7 ± 3.9	0.1893

Values are means ± SD. AMI, acute myocardial ischemia; LV, left ventricular; PVC, premature ventricular complex; VF + ve and VF − vs., animals that did (+) and did not (−) develop ventricular fibrillation, respectively.

### Myocardial Infarction Results in Sustained BVR Elevation

**Figure 5.**
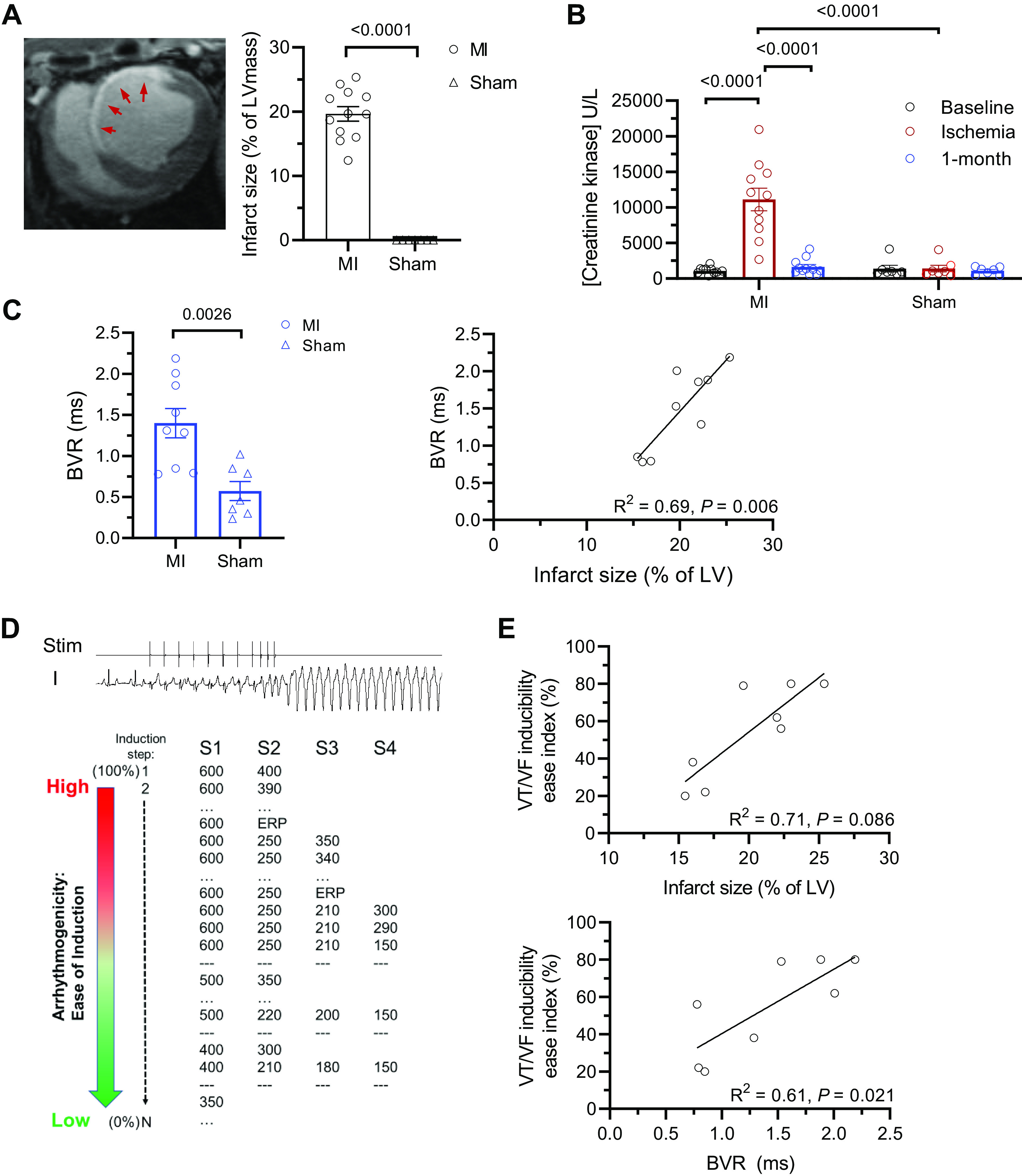
Beat-to-beat variation of repolarization (BVR) is elevated and associated with arrhythmia vulnerability in chronic myocardial infarction (MI). *A*, *left*: example of midventricular short-axis cMRI indicating 19.0% infarction (red arrows) after 1 mo. *A*, *right*: summary data of infarct size, *n* = 12 MI and 7 sham; two-tailed *t* test. *B*: serum creatinine kinase concentration at baseline, ischemia, and after 1 mo. Mixed-effects model ANOVA with Bonferroni posttest. *C*, *left*: summary data of BVR after 1 mo in MI (*n* = 9) and sham (*n* = 7); two-tailed *t* test. *C*, *right*: correlation between infarct size and BVR; *n* = 9 MI. Linear regression analysis. *D*: illustration of programmed stimulation protocol to induce ventricular arrhythmia (VT/VF; *top*) and the algorithm used to assess inducibility index based on aggressiveness of stimulation protocol (*bottom*). *E*, *top*: correlation between infarct size and VT/VF inducibility and bottom, correlation between BVR and VT/VF inducibility; *n* = 8 MI. Linear regression analysis. ANOVA, analysis of variance; LV, left ventricular; VF, ventricular fibrillation; VT, ventricular tachycardia; *n*, number of animals.

Finally, we investigated the long-term impact of MI on repolarization lability by assessing ECG BVR in *lead II* or *V4* as appropriate. Figure 5*A* illustrates the extent of infarction 1 mo after AMI, showing a 19.0% LV infarct, illustrated by a midventricular slice of the cMRI of an MI animal. BVR correlated with the infarct size (*R*^2^ = 0.69, *P* = 0.006). Serum CK increases during ischemia but returned to baseline values at 1 mo after AMI (11,116.45 ± 5,256.56 U/L vs. baseline, 1,074.0 ± 486.10 U/L vs. 1 mo, 1,634.45 ± 1,122.68 U/L, *P* < 0.0001) confirming absence of ongoing ischemia at this time point (Fig. 5*B*). One month after AMI, BVR under anesthesia was elevated in MI animals compared with sham (1.43 ± 0.50 ms vs. sham, 0.57 ± 0.30 ms, *P* = 0.009) (Fig. 5*C*). BVR in MI animals was lower after 1 mo than immediately post-AMI (AMI, 2.03 ± 0.86 ms vs. 1 mo, 1.47 ± 0.57 ms, *P* = 0.013), but did not normalize (baseline, 0.40 ± 0.20 ms vs. 1 mo, 1.47 ± 0.57 ms, *P* = 0.001). In addition, the effective refractory period (ERP), the shortest first extrasystole (S2) coupling interval at the 400 ms drive train that did not capture the ventricle was longer in MI animals (215 ± 30 ms vs. sham, 160 ± 10 ms, *P* = 0.009). This suggests an altered repolarization reserve and excitability.

We further tested the hypothesis that BVR could be used for the prediction of arrhythmia vulnerability, using PES to induce VT/VF and quantified the ease of induction (Fig. 5*D*). In all MI animals, VT/VF could be induced while only one in six shams was inducible (Supplemental Fig. S5). The inducibility of arrhythmia correlated with the infarct size (*R*^2^ = 0.71, *P* = 0.086) and the magnitude of baseline BVR after infarction (*R*^2^ = 0.67, *P* = 0.022).

## DISCUSSION

This study evaluated BVR during AMI in a preclinical animal model. The main findings were that BVR increased on the surface ECG during AMI and surged in the minute preceding VT/VF. In the chronic phase, BVR remained elevated compared with normal hearts and correlated with arrhythmogenicity.

### Spatial and Temporal Repolarization Variability during AMI

The use of this animal model offers the advantage of monitoring QT variability and possibly lethal arrhythmia during the entire period of ischemia. In the acute setting of ischemia, repolarization variability has been demonstrated both experimentally and in patients. Several studies demonstrated increased repolarization lability after myocardial infarction noninvasively by ECG analysis of QT variability (16, 30, 31) and invasively by assessment of BVR (21, 22) and TWA (19, 32). Normalized QT variability and QT variability index determined on 5-min epochs rose during ischemia in holters of patients with intermittent ST-T-segment changes (17). In a population presenting with ST-segment elevation myocardial infarction myocardial damage, measured as troponin rise on admission was associated with a significantly higher QT variability and more arrhythmia on reperfusion (18, 33). Our findings are congruent with and extend these observations. We demonstrated that BVR increased within the first 5 min of AMI in a spatial pattern congruent with ST deviation. Significant ST deviation was observed in II, aVF, and precordial leads V1-6, a pattern reflecting the extensive anteroseptal ischemia (34, 35). The pattern of BVR increase in the 12 leads had a remarkable similarity to ST patterns and a strong correlation to injury by cumulative scores (36). These findings are comparable to the fact that QT variability index in leads reflecting the site of infarct was correlated with the extent of LV damage and that patterns of TWA that were unique to regional injury reflected the regional repolarization vectors (19, 30). However, we did not observe significant changes in QT duration during AMI, although QT dispersion and BVR were increased. Together, these findings suggest an interplay between temporal and spatial lability in AMI.

The electrophysiological changes producing ST-segment changes during AMI are attributable to altered cellular electrophysiology and also underlie repolarization lability (37, 38). Potassium currents including K_ATP_ and voltage-gated K^+^ currents are modulated by increased extracellular potassium [K_o_], ATP depletion, pH changes, and ROS during ischemia. Calcium handling is also compromised manifesting characteristically as calcium alternans because of SERCA dysfunction and calcium-handling proteins. In addition, cell-to-cell uncoupling during AMI contributes to conduction abnormalities in local or regional differences and dispersion of repolarization (39).

### Temporal Changes in BVR Herald Imminent SCD during AMI

Arrhythmias occur frequently during AMI and a significant proportion are severe leading to SCD (2); however, factors predicting onset of VF in AMI continue to be investigated (4). In our study, VF was observed frequently at ∼20 min after onset of the AMI. Prior reports described two distinct arrhythmia phases in AMI: *phases 1a* (<10 min) and *1b* (15–30 min) (7, 39). The change in BVR in our study exhibited a similar pattern with a distinct increase in the early AMI phase. BVR increased significantly after 5 min of AMI as did the number of PVCs. In animals that developed VF, the increased BVR during AMI surged 1 min before VF, and more frequent PVCs were observed in this period. These findings suggest a distinct surge in BVR heralds arrhythmia during AMI, and that there may be a threshold of repolarization lability required for the occurrence of VF (9). Furthermore, we observed PVCs as the triggering event in our model. The coupling interval of PVCs initiating VT/VF tended to be shorter than those during early ischemia. These findings support recent reports of acute BVR increase preceding nsVT in patients with an implantable cardiac defibrillator on Holter monitoring (13, 14). However, contrasting findings have been reported by Sachdev et al. (40) who did not observe QT variability preceding arrhythmias in patients with AMI admitted to a coronary care unit. An important difference that may explain this difference is that QT variation was sampled at time intervals of 1 h, which could have made it possible to miss an acute temporary increase in repolarization lability.

Together, these data suggest an interplay between triggers and substrate in line with classical arrhythmia framework and the R from T phenomenon (28, 41).

### Increased BVR in Chronic MI and Arrhythmia Vulnerability

Temporal variability of repolarization has been extensively studied in chronic cardiac remodeling including the CAVB dog model (11, 42), the pig MI model (16, 43), and validated in clinical reports (25). In this model, BVR remained increased 1-mo post-AMI and correlated with infarct size, suggesting that the degree of remodeling may contribute to repolarization lability. An increased BVR reflects a reduced repolarization reserve (38) consistent with reduced repolarizing potassium currents and abnormal calcium handling in myocytes from chronic infarcted hearts (43, 44).

QT variability has been shown to be associated with an increased risk for lethal ventricular arrhythmia in patients with structural heart disease and after myocardial infarction (45–47). We observed a correlation between infarct size and arrhythmia inducibility in agreement with previous reports and likely still an important factor (29, 48). Recent studies have taken this further and advocated a role for detailed analysis of the infarct to be considered in risk assessment, but is yet to be incorporated into clinical practice guidelines (49, 50). We used a clinical PES protocol as a surrogate for arrhythmogenicity. In our model, monomorphic VT, considered to be specific, could be induced in all MI animals, but only in one sham-instrumented animal. The ERP of MI animals was longer suggesting a reduced excitability and conduction that facilitated conduction block and reentrant arrhythmias. The finding that VT inducibility was correlated to BVR suggests that repolarization lability may be a factor in the arrhythmia mechanism.

### Clinical Implications

The key finding of this study is that BVR increases during AMI and surges before arrhythmia onset. This suggests that differences in BVR should be examined in patients who do or do not develop ventricular arrhythmia during acute coronary syndromes and that monitoring BVR may be useful in the acute setting, for monitoring the risk of VF after AMI in the critical or coronary care unit settings. Beyond this, monitoring BVR may have value in implantable cardiac devices or wearables. The feasibility of this has been demonstrated in a dog model where the intracardiac electrogram was used to calculate BVR and detect and prevent imminent arrhythmia (51). Our data derived from a clinically relevant setting of AMI support the role of BVR monitoring even in the setting of dynamic T-wave changes, although further validation in humans is needed.

Arrhythmia and SCD risk stratification after MI remain challenging. Despite the fact that LVEF remains the most important parameter clinically used in this setting, electrophysiological parameters including inducible VT and spontaneous nsVT occurrence are important and already considered in risk stratification. The correlation between BVR and inducibility suggests that BVR may also be a noninvasive and possibly useful surrogate marker to be tested in this setting.

### Limitations

Some aspects of the specific AMI model employed in this study should be considered when interpreting the results. It is important to keep in mind that pigs are more vulnerable to ventricular arrhythmia than humans, possibly due to differences in the structure of their His-Purkinje network and in some specific currents (52). The use of general anesthesia may impact arrhythmogenesis as anesthetic drugs are known to influence arrhythmias. Moreover, all animals (including sham animals) were premedicated with amiodarone for 3 days to avoid excess mortality, the individual effect of amiodarone may be a confounder. In addition, AMI was induced in young healthy pigs, which is quite different from the clinical phenotype of IHD that occurs usually in older persons with comorbidities including obesity, diabetes, and hypertension. In addition, modulatory factors including sympathetic stimulation, mechanoelectrical feedback, and local electrolyte imbalance may contribute to initiation of VT/VF but were not investigated in this study.

We only assessed short-term variability of QT(STVQT), as a variable for beat-to-beat QT interval variability, whereas several other variables have been described (10). The analysis of BVR during AMI was also limited to defined time points. In an ideal situation, a continuous record of the sequential and dynamic changes in BVR would provide a complete picture. This should be addressed by future work on adaptive algorithms and machine learning to allow continuous monitoring of the QT and BVR.

The pattern of BVR dispersion observed in the 12-lead ECG was not validated with direct measurements of local and regional repolarization. Future work should evaluate the interplay between spatial and temporal variability in AMI with the use of mapping tools that were beyond the scope of this study.

### Conclusions

AMI induced temporal and spatial repolarization variability which was consistent with the extent and severity of injury. There was a distinct surge in BVR before arrhythmia onset, which identifies BVR as a possible useful parameter for monitoring and early warning systems. In the chronic MI setting, BVR remained elevated and increased reflecting a reduced repolarization reserve, which correlated to arrhythmia vulnerability suggesting BVR may also be useful in future arrhythmia risk stratification.

## DATA AVAILABILITY

Data will be made available upon reasonable request.

## SUPPLEMENTAL DATA

10.48804/QT0K2J Supplemental Figs. S1–S5: https://doi.org/10.48804/QT0K2J.

## GRANTS

M.A. was supported as doctoral researcher by the Fund for Scientific Research Flanders (FWO). R.W. and P.C. are supported by a KU Leuven Grant BOF-C1 (C14/18/079). R.W. is supported as senior clinical researcher by the FWO. This research received funding from the Flemish Government AI Research Program (to S.H. and J.M.). C.V. acknowledges financial support from the European Space Agency, BELSPO-NEPTUNE.

## DISCLOSURES

R.W. reports research funding from Abbott, Biotronik, Boston Scientific, Medtronic; speakers and consultancy fees from Medtronic, Boston Scientific, Biotronik, Abbott. None of the other authors has any conflicts of interest, financial or otherwise, to disclose.

## AUTHOR CONTRIBUTIONS

M.A., P.C., and R.W. conceived and designed research; M.A., S.I., J.M., D.W., A.T., and P.C. performed experiments; M.A., S.I., J.M., D.W., and A.T. analyzed data; M.A., S.I., J.M., D.W., A.T., P.C., and R.W. interpreted results of experiments; M.A. prepared figures; M.A. drafted manuscript; M.A., S.I., J.M., D.W., A.T., S.V.H., C.V., K.S., P.C., and R.W. edited and revised manuscript; M.A., S.I., J.M., D.W., A.T., S.V.H., C.V., K.S., P.C., and R.W. approved final version of manuscript.
